# Depressive symptoms and associated factors among travestis and transsexuals: a cross-sectional study

**DOI:** 10.1590/0034-7167-2023-0071

**Published:** 2024-07-15

**Authors:** Glauber Weder dos Santos Silva, Karina Cardoso Meira, Eder Samuel Oliveira Dantas, Sávio Marcelino Gomes, Iago Matheus Bezerra Pedrosa, Francisco Arnoldo Nunes de Miranda

**Affiliations:** I Secretaria de Saúde Pública do Estado do Rio Grande do Norte. Natal, Rio Grande do Norte, Brazil; II Universidade Federal do Rio Grande do Norte. Natal, Rio Grande do Norte, Brazil; III Universidade Federal de São Paulo. São Paulo, São Paulo, Brazil; IV Universidade Federal da Paraíba. João Pessoa, Paraíba, Brazil

**Keywords:** Transgender Persons, Sexual and Gender Minorities, Gender-Based Violence, Depression, Cross-Sectional Studies, Personas Transgénero, Minorías Sexuales y de Género, Violencia de Género, Depresión, Estudios Transversales

## Abstract

**Objectives:**

to estimate the prevalence of depressive levels and their associated factors among transvestite and transsexual individuals.

**Methods:**

this cross-sectional study involved 58 participants assisted by non-governmental organizations. The Beck Depression Inventory was utilized to assess levels of depression, complemented by a sociodemographic questionnaire and a questionnaire on experiences of violence. Data were analyzed using descriptive statistics and Poisson regression with robust variance.

**Results:**

a prevalence of 27.6% (95% CI = 11.50-39.10) for moderate to severe levels of depression was observed. This prevalence was associated with being unmarried (PR = 1.19; 95% CI = 1.10-1.28) and experiencing violence in healthcare services (PR = 2.30; 95% CI = 1.10-4.81).

**Conclusions:**

the absence of a partner and experiences of violence in healthcare settings negatively impacted mental health, leading to an increased prevalence of depressive symptoms among transvestite and transsexual individuals. Advocating for transgender rights and providing ongoing education in health care for professionals are critical strategies in promoting the mental health of this population.

## RESUMEN

**Objetivos:**

estimar la prevalencia de niveles depresivos y factores asociados entre personas travestis y transexuales.

**Métodos:**

estudio transversal con 58 participantes asistidos por organizaciones no gubernamentales. Se utilizó el Inventario de Depresión de Beck para la evaluación de los niveles depresivos y un cuestionario sociodemográfico y de experiencias de violencia. Los datos fueron analizados mediante estadística descriptiva y regresión de Poisson con varianza robusta.

**Resultados:**

se observó una prevalencia del 27,6% (IC95%=11,50-39,10) de niveles depresivos moderados a graves, asociada al estado civil soltero (RP=1,19; IC95%=1,10-1,28) y a la violencia en los servicios de salud (RP=2,30; IC95%=1,10-4,81).

**Conclusiones:**

vivir sin pareja y experimentar violencias en los servicios de salud perjudicaron la salud mental y aumentaron la prevalencia de síntomas depresivos entre personas travestis y transexuales. La defensa de los derechos de las personas trans y la educación permanente en salud para los profesionales son estrategias esenciales para promover la salud mental de esta población.

## INTRODUCTION

Depression and its symptoms arise or develop uniquely among individuals and social groups. Among travesti and transsexual individuals, herein referred to as trans individuals, there is a higher risk of being victims of violent acts, and challenges in accessing employment, income, health services, and education are closely linked to depression^(1-3)^. Trans individuals are those who identify with a gender different from that assigned at birth^(3-4)^. These identities are self-determined and self-reported, standing in contrast to hegemonic and heterocisnormative gender roles^(1-4)^.

Trans individuals exhibit higher rates of depression compared to the cisgender population (those who identify with the gender assigned to them at birth)^(5-7)^. According to the Gender Minority Stress Theory (GMST)^(8-9)^, biological and social mechanisms interact synergistically to determine health disparities in trans individuals. This group experiences higher levels of social stress than cisgender individuals. Consequently, prolonged high levels of cortisol, coupled with experiences of social rejection, violence, and psychoactive substance use, contribute to mental health issues like anxiety and depression^(10)^.

In this context, Brazil’s structural sociocultural factors may exacerbate mental health problems within this population group^(11)^. The nation faces high rates of violence against transgender individuals^(12)^, with a significant prevalence of such violence reported in the Information System for Notifiable Diseases (SINAN)^(13)^. Moreover, there has been an increasing trend in homicides against the Lesbian, Gay, Bisexual, Travesti, Transsexual, Queer, Intersex, Asexual, and other gender variabilities (LGBTQIAP+) population from 2002 to 2016^(14)^.

In Brazil, a significant milestone for trans population health is the establishment of the National Policy for the Comprehensive Health of Lesbians, Gays, Bisexuals, Travesti, and Transsexuals (PNSI-LGBT)^(15)^. This policy guides care actions and organizes the Transsexualizing Process within the Unified Health System. Despite these advances, the considerable challenges in securing health access for the trans population necessitate research into themes that could enhance their health status.

Therefore, studies tracking depressive symptoms and their associated factors in the trans population are vital for developing methodological strategies, appropriate health actions, and targeted care strategies for this group. This raises the research question: What is the prevalence of depressive symptoms and their associated factors among travestis and transsexuals?

## OBJECTIVES

To estimate the prevalence of depressive levels and associated factors among transgender and travesti individuals.

## METHODS

### Ethical Aspects

The study adhered to the ethical standards of Resolution 466/2012 of the National Health Council and received approval from the Human Research Ethics Committee of the Federal University of Rio Grande do Norte on March 8, 2019. Informed consent was obtained from all participants involved in the study in written form.

### Design, Period, and Location of Study

This cross-sectional study, guided by the STrengthening the Reporting of OBservational studies in Epidemiology (STROBE) tool, aimed to analyze suicidal ideation and depression among self-declared transgender and travesti individuals. It was conducted in the only four Non-Governmental Organizations (NGOs) for transgender rights in the state of Rio Grande do Norte, Brazil. These NGOs, the settings of this study, are located in two microregions of Rio Grande do Norte and have members statewide. They were founded and organized by the regional trans movement, advocating for social recognition and civil rights, namely: the Potiguar Association of Travestis and Transsexuals in Action for Consistency in Rio Grande do Norte; the Association of Travestis Finding Value and Engagement in Santa Cruz Health; the Association of Potiguar Trans Men; and the Association of Travestis Rediscovering Life. Data collection occurred from April to June 2019.

### Population or Sample; Inclusion and Exclusion Criteria

The study was conducted as a census and included individuals aged 18 years or older who self-identified as a transsexual or travesti (N=78). Excluded were candidates who joined any of the NGOs after the start of data collection, those not residing in Rio Grande do Norte, and intersex individuals or those self-identified with other gender variabilities (n=20). The study consisted of 58 subjects who met the eligibility criteria.

### Study Protocol

Initially, a questionnaire developed by the authors was used, covering sociodemographic variables (age, gender identity, sexual orientation, marital status, education level, religiosity, race/skin color, and income) and history of violence (type of violence experienced, relationship with the aggressor, and location of the violence). The form created by the authors was pre-tested with five local coordinators of the NGOs, who did not participate in the final sample.

Regarding the depression assessment instrument, the Beck Depression Inventory (BDI) was chosen. The Beck Scales are considered the “gold standard” for measuring the variables of interest and were validated in Portuguese in 2001 for both clinical and non-clinical use^(16)^. This self-report scale identifies the intensity of depressive symptoms in psychiatric and non-psychiatric populations, without diagnostic intent^(16)^. The BDI comprises 21 items, each offering four alternatives scored from 0 to 3 points, representing increasing degrees of severity of depressive symptoms. The following parameters were used for stratifying symptoms into depressive levels: minimal (0 to 11 points), mild (12 to 19 points), moderate (20 to 35 points), and severe (36 to 63 points)^(16)^. In this study population, a Cronbach’s alpha coefficient of 0.93 was obtained, indicating good internal consistency.

### Analysis of Results and Statistics

The data were initially analyzed with descriptions of absolute and relative frequencies for qualitative variables, and median and interquartile range for quantitative variables. The existence of statistically significant differences between the dependent variable (depressive levels) and other independent variables was evaluated using the chi-square test or Fisher’s exact test depending on data suitability, likelihood ratio, and the Student’s t-test or Kruskal-Wallis test depending on the normality of the data. A 5% confidence level was used.

Multiple analyses were conducted using a Poisson regression model with robust variance. Variables significant at the 20% level in the bivariate analysis were candidates for the final model. Models were compared using the Akaike Information Criterion (AIC)^(17)^. The final model considered statistical significance at the 5% level, as well as biological and epidemiological plausibility, estimating associations based on adjusted Prevalence Ratios and 95% confidence intervals. All analyses were performed using the R statistical software, version 4.2.0.

## RESULTS

In the study sample, a higher proportion of transgender individuals was observed (62.1%), with a median age of 24.50 years (IQR=11.25 years), living with a partner (81.0%), educated up to high school level (77.6%), of African descent (69.0%), earning up to one minimum wage (60.3%), and the majority did not have a fixed income (82.8%). A prevalence of 27.6% (95% CI=11.50-39.10) of individuals was found to be classified with moderate/severe depressive levels (Table 1).

**Table 1:** Sociodemographic characterization variables of travestis and transgender individuals assisted by non-governmental organizations (n=58), Natal, Rio Grande do Norte, Brazil, 2023

Sociodemographic Variables	n (%)
Gender Identity	
Travesti	22 (37.9)
Transsexual	32 (61.1)
Sexual Orientation	
Heterosexual	48 (82.7)
NonHeterosexual	10 (17.2)
Marital Status	
Living with Partner	47 (81.0)
Single	11 (19.0)
Religiosity	
Yes	37 (63.8)
No	21 (36.2)
Education Level	
Up to High School	45 (77.6)
Higher Education	13 (22.4)
Race/Skin Color	
White	18 (31.0)
Black	40 (69.0)
Fixed Income Source	
Yes	10 (17.2)
No	48 (82.8)
Monthly Income (Minimum Wage)	
Up to 1	35 (60.3)
More than 1	23 (39.7)
Engagement in Sex Work	
Yes	17 (29.3)
No	41 (70.7)
Depressive Levels	
Minimal and Mild	42 (72.4)
Moderate and Severe	16 (27.6)

Nearly all participants reported experiencing violence (96.6%), with verbal violence being the most frequent (84.5%), followed by sexual violence (58.6%). The most common location for these occurrences was in public spaces (74.1%), with perpetrators being equally likely to be known or unknown individuals (Table 2).

**Table 2:** Characterization variables of the violence experience of travestis and transgender individuals assisted by non-governmental organizations (n=58), Natal, Rio Grande do Norte, Brazil, 2023

Victimization Variables	n (%)
Experienced Any Type of Violence?	
Yes	56 (96.60)
No	2 (3.40)
Physical Violence	
Yes	23 (39.70)
No	35 (60.30)
Verbal Violence	
Yes	49 (84.50)
No	9 (15.50)
Sexual Violence	
Yes	34 (58.60)
No	24 (41.40)
Location of Violence - Home	
Yes	21 (36.20)
No	37 (63.80)
Location of Violence - Street	
Yes	43 (74.10)
No	15 (25.90)
Location of Violence - School	
Yes	25 (43.10)
No	33 (56.90)
Location of Violence - Health Services	
Yes	7 (12.10)
No	51 (87.90)
Location of Violence - Other Public and Private Services	
Yes	6 (10.30)
No	52 (89.70)
Family Member as Perpetrator	
Yes	21 (36.20)
No	37 (63.80)
Relationship with the Perpetrator?	
Known	22 (37.90)
Unknown	21 (36.20)
Unknown and Known	13 (22.40)
Did Not Experience Violence	2 (3.40)

Regarding the classification of individuals according to depressive levels (minimal/mild and moderate/severe) and sociodemographic variables, a statistically significant difference was found only in the marital status variable, where all single individuals were classified with a moderate/severe depressive level (p=0.020) (Table 3). There was no observed difference in the median age of individuals with moderate/severe depressive levels compared to those classified with minimal/mild depressive levels (24.0 (IQR=15.12) vs. 24.50 (IQR=12.50), p=0.889).

**Table 3:** Distribution of Sociodemographic Variables According to Depressive Levels of Travestis and Transgender Individuals Assisted by Non-Governmental Organizations (n=58), Natal, Rio Grande do Norte, Brazil, 2023

Variables	Depressive Levels		*p* value
	Minimal/Mild n (%)	Moderate/Severe n (%)	
Gender Identity			
Travesti	14 (63.6)	8 (36.3)	0.190^*^
Transsexual	28 (77.78)	8 (22.22)	
Sexual Orientation			
Heterosexual	35 (72.22)	7 (38.88)	0.573^†^
NonHeterosexual	13 (81.25)	3 (18.75)	
Marital Status			
Living with Partner	11 (26.19)	31 (73.81)	0.021^*^
Single	0 (0.00)	16 (100.00)	
Religiosity			
Yes	27 (64.29)	15 (35.71)	0.566^*^
No	10 (62.50)	6 (37.50)	
Education Level			
Up to High School	2 (12.50)	14 (87.50)	0.233^*^
Higher Education	31 (73.80)	11 (26.20)	
Race/Skin Color			
White	10 (55.56)	8 (44.44)	0.061^*^
Black/Mixed	32 (80.00)	8 (20.00)	
Employability			
Yes	34 (70.83)	14 (29.17)	0.446^*^
No	8 (80.00)	2 (20.00)	
Atua como profissional do sexo??			
Sim	32 (78.05)	9 (21.95)	0.120^*^
Não	10 (58.82)	7 (41.18)	
Renda mensal (salário-mínimo)			
Até 1	17 (73.91)	6 (26.09)	0.546^*^
> 1	25 (71.43)	10 (28.57)	

* Teste Qui-quadrado;

† Teste Exato de Fisher.

A higher proportion of moderate/severe depressive levels was observed in individuals who reported experiencing violence in healthcare settings (p=0.030) (Table 4).

**Table 4:** Violence Against Travestis and Transgender Individuals According to Depressive Levels (n=58), Natal, Rio Grande do Norte, Brazil, 2023

Violence	Depressive Levels		*p* value
	Minimal/Mild n (%)	Moderate/Severe n (%)	
Experienced Violence			
Yes	41 (73.21)	15 (26.79)	0.488^*^
No	1 (50.00)	1 (50.00)	
Physical Violence			
Yes	17 (73.91)	6 (26.09)	0.845^*^
No	25 (71.43)	10 (28.57)	
Verbal Violence			
Yes	35 (71.43)	14 (28.57)	0.655^*^
No	7 (77.78)	2 (22.22)	
Sexual Violence			
Yes	22 (64.71)	12 (35.29)	0.121^‡^
No	20 (83.33)	4 (16.67)	
Psychological Violence			
Yes	18 (66.67)	9 (33.33)	0.363^*^
No	24 (77.42)	7 (22.58)	
Number of Types of Violence Experienced			
Up to 1	15 (93.75)	1 (6.25)	0.253^‡^
2 or More	27 (64.29)	15 (35.71)	
Location of Violence - Home			
Yes	15 (71.43)	6 (28.57)	0.891^*^
No	27 (72.97)	10 (27.03)	
Location of Violence - Street			
Yes	29 (67.44)	14 (32.56)	0.159^*^
No	13 (86.67)	2 (13.33)	
Location of Violence - School			
Yes	18 (72.00)	7 (28.00)	0.952^*^
No	24 (72.73)	9 (27.27)	
Location of Violence - Health Services			
Yes	3 (42.86)	4 (57.14)	0.030^†^
No	39 (76.47)	12 (23.53)	
Location of Violence - Other Public and Private Services			
Yes	4 (66.67)	2 (33.33)	0.660^*^
No	38 (73.08)	14 (26.92)	
Family Member as Perpetrator			
Yes	15 (71.43)	6 (28.57)	0.891^†^
No	27 (72.97)	10 (27.03)	
Relationship with the Perpetrator?			
Known	16 (72.73)	6 (27.27)	0.251^‡^
Unknown	17 (80.95)	4 (19.05)	
Unknown and Known	7 (53.85)	6 (46.15)	
Did Not Experience Violence	2 (100.00)	0 (0.00)	

* Chi-square Test;

† Fisher's Exact Test;

‡ Likelihood Ratio.

In the multiple analysis, the variables that remained associated with depressive levels were marital status and experience of violence in healthcare settings. Participants who were single showed a higher prevalence of depressive levels (moderate/severe) compared to those living with a partner (PR=1.19). Similarly, a higher prevalence of moderate/severe depressive levels (PR=2.30) was also observed in those who reported experiencing violence in healthcare settings (Table 5).

**Table 5:** Multiple Regression Model on the Association Between Depressive Levels, History of Violence, and Marital Status Among Travestis and Transgender Individuals Assisted by Non-Governmental Organizations in the State of Rio Grande do Norte, Natal, Rio Grande do Norte, Brazil, 2023

Variables	Crude RP^*^ (95% CI^†^)	Adjusted RP (95% CI)
Marital Status		
Living with Partner	1^‡^	
Single	1.27 (1.19-1.76)	1.19 (1.10-1.28)
Location of Violence - Healthcare Service		
No	1^‡^	
Yes	1.30 (1.03-1.79)	2.30 (1.10-4.81)

* RP - Prevalence Ratio/Adjusted Prevalence Ratio;

† CI - Confidence Interval;

‡ Reference Category.

## DISCUSSION

This research revealed a high prevalence of moderate to severe depressive levels among transgender individuals and travestis assisted by Non-Governmental Organizations (NGOs) in Rio Grande do Norte, Brazil. This prevalence was associated with marital status and experiences of violence in the context of health services.

The high prevalence (27.6%) of moderate to severe depressive symptoms in the 15 days prior to data collection was greater than that found in studies conducted in Cambodia^(5)^, where 21% of participants displayed severe symptoms of depression, and in Brazil^(13)^, where 16.0% experienced severe depression. These findings might be influenced by differences in the measurement instruments used for the variables. Nonetheless, the severity of depressive levels in the lives of travestis and transgender individuals across different regions is striking.

The prevalence of depressive symptoms in the transgender population is significantly higher compared to their cisgender counterparts, with a prevalence ratio 1.03 times higher for the development of depression and 2.53 times higher for attempted suicide, compared to cisgender individuals^(18)^.

These disparities can be explained by the Gender Minority Stress Theory, which posits that social minorities experience specific societal stressors, in addition to everyday stressors. In this context, transgender individuals encounter a high prevalence of stressors such as harassment, trauma, rejection, and gender-related abandonment, along with the numerous acts of violence they face in society, often manifested through transphobia^(8,19)^.

The occurrence of depression among travestis and transgender individuals appears to be a consequence of a life context where the debate on gender and sexuality issues is restricted, despite being a growing discussion. The adverse effects are evident in the difficulty of social integration, in intrafamily relationships, and in the lack of opportunities in the job market^(5)^. In this setting, the present study indicated that the majority of travestis and transgender individuals earn up to a minimum wage and lack a steady income. Along with limited access to work and income, participants in this study reported a high incidence of violent experiences (96.60%), particularly verbal and sexual violence, which may be linked to the high prevalence of moderate to severe depressive symptoms observed in this population.

Violence directed at transgender bodies is ingrained in the Brazilian social structure and is linked to machismo and heterocisnormativity, which operate insidiously and through logics that hierarchize bodies and lives. Within this framework, transgender individuals are rendered vulnerable and oppressed for not adhering to established hegemonic norms. Under this same system, instances of murder, rape, physical and verbal assault, psychological and sexual abuse, and attacks in public or private spheres, including health services and educational institutions, can occur^(20)^.

Regarding compromised social relationships, our study found that single travestis and transgender individuals are more susceptible to developing moderate to severe depressive symptoms compared to those with partners. This reflects the challenge travestis and transgender individuals face in forming stable relationships, as they are often marginalized in emotional connections. It is important to recognize that human affectivity is closely linked to mental health, and marginalization in this realm can lead to self-esteem and self-concept issues, which may manifest as depressive symptoms^(21)^.

Conversely, measures that support the recognition of gender identities, incorporate gender education in the educational system, and promote familial and social acceptance, serve as strategies to enhance the sociability of travestis and transgender individuals and to safeguard their mental health. Social support, characterized by positive conjugal/affective relationships, is acknowledged as a protective factor against suicidal ideation. This support is augmented by peer experiences, the availability of health care services, disciplinary consistency, and family cohesion^(22-23)^.

Experiences of violence in health services have been linked to a higher prevalence of moderate to severe depressive symptoms. The lack of preparedness among health professionals to address sexual and gender diversity is extensively debated in the literature. Trans individuals often report that their needs are not fully met, citing inadequate care or experiencing prejudice from health care staff members^(24-26)^. Multiple studies document this issue, indicating that violence by health professionals is a significant risk factor for depression^(5,26-27)^.

Another concern, related to the high prevalence of depressive symptoms in the trans population, is suicidal behavior. Although not the focus of this study, it is critical to recognize that both suicidal ideation and attempts are prevalent among travestis and transgender individuals. Suicidal ideation is commonly associated with moderate and severe depression in this population^(28)^.

Therefore, it is understood that beyond depressive symptoms and their associated factors, travestis and transgender individuals are at risk of both voluntary and involuntary deaths when exposed to violence in various social, institutional, and family contexts. In the Brazilian context, this reality contradicts the PNSI-LGBT^(15)^. This policy aims to educate health professionals on the rights of these populations, employing strategies for ongoing health education and specialized services to provide appropriate care and address mental health challenges.

### Study limitations

This study presents several limitations that should be taken into account when interpreting the results. First, its cross-sectional nature prohibits the inference of causal relationships between the factors studied and the observed levels of depression. Additionally, the sample, recruited from only four Non-Governmental Organizations, limits the generalizability of the findings to other regions and contexts. Another significant factor is the reliance on self-reported data, which may be subject to memory biases and the desire to present oneself in a favorable light. Furthermore, the study’s setting within NGOs and the collective identity of the organizations might have had a protective effect against the development of depressive symptoms. It is conceivable that the prevalence of depression among transgender and travesti individuals not assisted by NGOs could be even more distressing. Finally, the instruments used to measure depressive symptoms may not capture the full complexity and subtleties of this population’s mental health experiences, especially within Brazil’s culturally diverse context.

### Contributions to Nursing, Health, or Public Policy

This study contributes to the health sector by focusing on a key and socially vulnerable population in the process of mental health deterioration. The insights into this population’s health status can guide health professionals in recognizing factors related to minority stress, employing tools and social mechanisms for health protection and preservation, and advocating for the rights of transgender and travesti individuals in public and legislative arenas. Moreover, the PNSI-LGBT faces the challenge of enhancing the delivery of care to reduce mental health issues, address violence in health services, and promote mental well-being for transgender and travesti people. Thus, the insights from this study have the potential to positively impact various aspects of Nursing, public health, and policy-making, fostering a more inclusive and equitable society.

## CONCLUSIONS

We have identified high levels of depression (moderate to severe) in the population of transgender and travesti individuals served by non-governmental organizations, correlated with marital status and experiences of violence in health services. Our study highlights the need for stronger public policies and professional training programs that prioritize diversity, inclusion, and cultural competence. It also reaffirms the necessity for advocacy and social support efforts to combat the marginalization, discrimination, and violence these individuals endure. We recommend further Brazilian research employing different methodological approaches, such as qualitative, theoretical, or population-based studies, to generate additional evidence levels.
