# Supplementary material for: Depressive symptoms and associated factors among travestis and transsexuals: a cross-sectional study
Source: Rev Bras Enferm. 2024 Jul 15;77(Suppl 3):e20230071. doi: 10.1590/0034-7167-2023-0071 (PMC11253834; doi:10.1590/0034-7167-2023-0071)
Supplement: Translation

# Translation: Sintomas depressivos e fatores associados entre travestis e transexuais: estudo transversal

**DOI:** 10.1590/0034-7167-2023-0071pt

**Authors:** Glauber Weder dos Santos Silva, Karina Cardoso Meira, Eder Samuel Oliveira Dantas, Sávio Marcelino Gomes, Iago Matheus Bezerra Pedrosa, Francisco Arnoldo Nunes de Miranda

**Affiliations:** I Secretaria de Saúde Pública do Estado do Rio Grande do Norte. Natal, Rio Grande do Norte, Brasil; II Universidade Federal do Rio Grande do Norte. Natal, Rio Grande do Norte, Brasil; III Universidade Federal de São Paulo. São Paulo, São Paulo, Brasil; IV Universidade Federal da Paraíba. João Pessoa, Paraíba, Brasil

**Keywords:** Pessoas Transgênero, Minorias Sexuais e de Gênero, Violência de Gênero, Depressão, Estudos Transversais

## Abstract

**Objetivos:**

estimar a prevalência de níveis depressivos e fatores associados entre pessoas travestis e transexuais.

**Métodos:**

estudo transversal com 58 participantes assistidos por organizações não governamentais. Utilizou-se o *Beck Depression Inventory* para avaliação dos níveis depressivos e um questionário sociodemográfico e de experiências de violência. Os dados foram analisados por meio de estatística descritiva e regressão de Poisson com variância robusta.

**Resultados:**

observou-se uma prevalência de 27,6% (IC95%=11,50-39,10) de níveis depressivos moderados a graves, associada ao estado civil solteiro (RP=1,19; IC95%=1,10-1,28) e à violência nos serviços de saúde (RP=2,30; IC95%=1,10-4,81).

**Conclusões:**

viver sem companheiro(a) e experienciar violências nos serviços de saúde prejudicaram a saúde mental e aumentaram a prevalência de sintomas depressivos entre pessoas travestis e transexuais. A defesa dos direitos de pessoas trans e a educação permanente em saúde para profissionais são estratégias essenciais para promover a saúde mental dessa população.

## INTRODUÇÃO

A depressão e seus sintomas surgem ou se desenvolvem de maneira singular nos indivíduos e grupos sociais. Entre as pessoas travestis e transexuais, aqui denominadas de pessoas trans, o maior risco de serem vítimas de atos violentos e a dificuldade de acesso ao emprego, renda, aos serviços de saúde e educação apresentam estreita relação com a depressão^(1-3)^. Pessoas Trans são aquelas que reivindicam para si uma identidade de gênero diferente da designada ao nascimento^(3-4)^. São identidades de gênero autodeterminadas e autorrelatadas, em oposição aos papéis de gênero hegemônicos e heterocisnormativos^(1-4)^.

Pessoas trans apresentam prevalências mais elevadas de depressão comparadas à população cisgênera (pessoas que se identificam com o gênero ao qual foram atribuídas no nascimento)^(5-7)^. Segundo a *Gender Minority Stress Theory* (GMST)^(8-9)^, mecanismos biológicos e sociais interagem de forma sinérgica na determinação das desigualdades em saúde de pessoas trans. Esta parcela da população é exposta a níveis de estresse social mais elevados do que as pessoas cisgêneras, assim, os altos níveis de cortisol a longo prazo, em conjunto com experiências de rejeição social, violências e uso de substâncias psicoativas, contribuem para o desenvolvimento de problemas de saúde mental, como ansiedade e depressão^(10)^.

Nesse sentido, o Brasil possui fatores socioculturais estruturais que podem contribuir para importantes problemas de saúde mental neste grupo populacional^(11)^, pois somos uma nação com elevados índices de violência contra pessoas transgêneras^(12)^, com alta prevalência de violência notificada no Sistema de Informação de Agravos de Notificação (SINAN)^(13)^. Além disso, apresentamos uma tendência ascendente de homicídios contra a população Lésbica, Gay, Bissexual, Travesti, Transexual, Queer, Intersexo, Assexuada e outras variabilidades de gênero (LGBTQIAP+) no período de 2002 a 2016^(14)^.

No Brasil, um grande marco no campo da saúde da população trans é a criação da Política Nacional de Saúde Integral de Lésbicas, Gays, Bissexuais, Travestis e Transexuais (PNSI-LGBT)^(15)^, responsável por orientar ações de cuidado e ordenar o Processo Transexualizador no âmbito do Sistema Único de Saúde. Ainda assim, diante dos enormes desafios na consolidação do acesso à saúde por parte da população trans, faz-se necessário estudar temáticas que possam contribuir com a melhoria do status de saúde dessas pessoas.

Desse modo, entende-se que realizar estudos que rastreiem sintomas depressivos e fatores associados na população trans pode colaborar com a construção de estratégias metodológicas, ações de saúde apropriadas e estratégias de cuidado voltadas para o grupo em questão. Dado o problema de pesquisa, questiona-se: qual é a prevalência de sintomas depressivos e fatores associados entre travestis e transexuais?

## OBJETIVOS

Estimar a prevalência de níveis depressivos e fatores associados entre pessoas travestis e transexuais.

## MÉTODOS

### Aspectos Éticos

O estudo atendeu às normas éticas da Resolução 466/2012 do Conselho Nacional de Saúde e obteve aprovação do Comitê de Ética em Pesquisa com Seres Humanos da Universidade Federal do Rio Grande do Norte em 08/03/2019. O Consentimento Livre e Esclarecido foi obtido de todos os participantes envolvidos no estudo por meio escrito.

### Desenho, Período e Local de Estudo

Estudo transversal, norteado pela ferramenta *STrengthening the Reporting of OBservational studies in Epídemiology* (STROBE), que intencionou analisar a ideação suicida e a depressão entre pessoas autodeclaradas travestis e transexuais, realizado nas quatro únicas Organizações Não Governamentais (ONGs) de direitos transexuais existentes no estado do Rio Grande do Norte, RN, Brasil. As ONGs, cenários deste estudo, estão sediadas em duas microrregiões do Rio Grande do Norte e possuem membros em todo o território estadual. Foram fundadas e organizadas pelo movimento regional de pessoas trans, na luta pelo reconhecimento social e direitos civis, a saber: Associação Potiguar de Travestis e Transexuais na Ação pela Coerência no Rio Grande do Norte; a Associação das Travestis Encontrando a Valorização e Atuação na Saúde Santa-Cruzense; a Associação de Homens-Trans Potiguares; e a Associação de Travestis Reencontrando a Vida. A coleta de dados ocorreu entre abril e junho de 2019.

### População ou Amostra; Critérios de Inclusão e Exclusão

O estudo configurou-se como um censo e incluiu sujeitos com idade igual ou superior a 18 anos, que se autoidentificavam como pessoa transexual ou travesti (N=78). Foram excluídos os candidatos que se associaram a alguma das ONGs após o início da coleta de dados, aqueles que não estavam residindo no Rio Grande do Norte, e pessoas intersexuais ou autoidentificadas com outras variabilidades de gênero (n=20). O estudo compôs-se de 58 sujeitos que atenderam aos critérios de elegibilidade.

### Protocolo de Estudo

Primeiro, utilizou-se um questionário elaborado pelos autores com variáveis sociodemográficas (idade, identidade de gênero, orientação sexual, estado civil, nível de escolaridade, religiosidade, raça/cor da pele e renda) e histórico de violência (tipo de violência sofrida, relação com o agressor e espacialidade da violência). O formulário elaborado pelos autores foi pré-testado com cinco coordenadores locais das ONGs, que não integraram a amostra final.

Em relação ao instrumento de avaliação da depressão, optou-se pelo Inventário de Depressão de Beck (BDI). As Escalas Beck são consideradas “padrão ouro” para a mensuração das variáveis de interesse em tela, as quais foram validadas para o português em 2001 para uso clínico e não clínico^(16)^. Esta escala é de autorrelato e identifica a intensidade de sintomas depressivos em populações psiquiátricas e não psiquiátricas, sem intenção psicodiagnóstica^(16)^. O BDI é composto por 21 itens, cada um contendo quatro alternativas pontuadas de 0 a 3 pontos, representando graus crescentes de gravidade de sintomas depressivos. Utilizou-se o seguinte parâmetro para a estratificação de sintomas em níveis depressivos: mínimo (0 a 11 pontos), leve (12 a 19 pontos), moderado (20 a 35 pontos) e grave (36 a 63 pontos)^(16)^. Na população deste estudo, obteve-se um coeficiente de *alfa* de Cronbach de 0,93, o que indicou boa consistência interna.

### Análise dos resultados e estatística

Os dados foram analisados, inicialmente, com descrição das frequências absolutas e relativas para variáveis qualitativas e mediana e intervalo interquartil para as variáveis quantitativas. Avaliou-se a existência de diferença estatisticamente significativa entre a variável dependente (níveis depressivos) e as demais variáveis independentes por meio do teste qui-quadrado ou teste exato de Fisher de acordo com adequabilidade dos dados, razão de verossimilhança e teste T de *Student* ou *Kruskal-Wallis Test* a depender da normalidade dos dados. O nível de confiança empregado foi de 5%.

A análise múltipla foi realizada pelo modelo de regressão de Poisson com variância robusta. Foram candidatas ao modelo final as variáveis significantes ao nível de 20% na análise bivariada. Os modelos foram comparados via Critério de Informação de Akaike (AIC)^(17)^. O modelo final considerou a significância estatística ao nível de 5%, plausibilidade biológica e epidemiológica, estimando as associações com base nas Razões de Prevalência ajustadas e intervalos de confiança de 95%. Todas as análises foram realizadas no software estatístico R versão 4.2.0.

## RESULTADOS

Na amostra do estudo, observou-se maior proporção de pessoas transexuais (62,1%), com mediana de 24,50 anos (IQR=11,25 anos), vivendo com companheiro (81,0%), com escolaridade até o ensino médio (77,6%), afrodescendente (69,0%), com renda de até 1 salário-mínimo (60,3%) e a maior frequência destes não apresentavam renda fixa (82,8%). Verificou-se prevalência de 27,6% (IC95%=11,50-39,10) de indivíduos classificados com níveis depressivos moderado/grave (Tabela 1).

**Tabela 1:** Variáveis de caracterização sociodemográfica de travestis e transexuais assistidas por organização não governamentais (n=58), Natal, Rio Grande do Norte, Brasil, 2023

Variáveis sociodemográficas	n (%)
Identidade de gênero	
Travesti	22 (37,9)
Transexual	32 (61,1)
Orientação sexual	
Heterossexual	48 (82,7)
Não Heterossexual	10 (17,2)
Estado civil	
Vivendo com companheiro	47 (81,0)
Solteiro	11 (19,0)
Possui religiosidade	
Sim	37 (63,8)
Não	21 (36,2)
Nível de escolaridade	
Até Ensino Médio	45 (77,6)
Ensino Superior	13 (22,4)
Raça/cor	
Branca	18 (31,0)
Negra	40 (69,0)
Fonte de renda fixa	
Sim	10 (17,2)
Não	48 (82,8)
Renda mensal (salário-mínimo)	
Até 1	35 (60,3)
>1	23 (39,7)
Atua como profissional do sexo?	
Sim	17 (29,3)
Não	41 (70,7)
Níveis depressivos	
Mínima e leve	42 (72,4)
Moderada e grave	16 (27,6)

Quase a totalidade dos participantes relataram ter sofrido violência (96,6%), sendo a violência verbal a de maior frequência (84,5%), seguida da violência sexual (58,6%), tendo como local de ocorrência a espacialidade da rua (74,1%), sendo os perpetradores em frequência semelhante, pessoas conhecidas e desconhecidas (Tabela 2).

**Tabela 2:** Variáveis de caracterização da experiência de violência de travestis e transexuais assistidas por organização não governamentais (n=58), Natal, Rio Grande do Norte, Brasil, 2023

Variáveis vitimização	n (%)
Já sofreu algum tipo de violência?	
Sim	56 (96,60)
Não	2 (3,40)
Violência física	
Sim	23 (39,70)
Não	35 (60,30)
Violência verbal	
Sim	49 (84,50)
Não	9 (15,50)
Violência sexual	
Sim	34 (58,60)
Não	24 (41,40)
Espacialidade da violência - Casa	
Sim	21 (36,20)
Não	37 (63,80)
Espacialidade da violência - Rua	
Sim	43 (74,10)
Não	15 (25,90)
Espacialidade da violência - Escola	
Sim	25 (43,10)
Não	33 (56,90)
Espacialidade da violência - Serviços de saúde	
Sim	7 (12,10)
Não	51 (87,90)
Espacialidade da violência - outros serviços púbicos e privados	
Sim	6 (10,30)
Não	52 (89,70)
Agressor familiar	
Sim	21 (36,20)
Não	37 (63,80)
Relação com o agressor?	
Conhecido	22 (37,90)
Desconhecido	21 (36,20)
Conhecido e desconhecido	13 (22,40)
Não sofreu violência	2 (3,40)

No que diz respeito à classificação dos indivíduos segundo níveis depressivos (mínimo/leve e moderado/grave) e as variáveis sociodemográficas, verificou-se diferença estatisticamente significativa somente com a variável estado civil, em que todos os indivíduos solteiros foram classificados com nível depressivo moderado/grave (p=0,020) (Tabela 3). Não se observou diferença entre a mediana da idade dos indivíduos que apresentaram nível depressivo moderado/grave em relação aos que foram classificados com nível depressivo mínimo/leve (24,0 (IQR=15,12) vs 24,50 (IQR=12,50), p=0,889).

**Tabela 3:** Distribuição das variáveis sociodemográficas segundo níveis depressivos de travestis e transexuais assistidas por organização não governamentais (n=58), Natal, Rio Grande do Norte, Brasil, 2023

Variáveis	Níveis depressivos		Valor de *p*
	Mínima/ leve n (%)	Moderado/Grave n (%)	
Identidade de gênero			
Travestis	14 (63,6)	8 (36,3)	0,190^*^
Transexuais	28 (77,78)	8 (22,22)	
Orientação sexual			
Heterossexual	35 (72,22)	7 (38,88)	0,573^†^
Não heterossexual	13 (81,25)	3 (18,75)	
Estado civil			
Vivendo com companheiro	11 (26,19)	31 (73,81)	0,021^*^
Solteiro	0 (0,00)	16 (100,00)	
Religiosidade			
Sim	27 (64,29)	15 (35,71)	0,566^*^
Não	10 (62,50)	6 (37,50)	
Escolaridade			
Até ensino médio	2 (12,50)	14 (87,50)	0,233^*^
Ensino superior	31 (73,80)	11 (26,20)	
Raça/cor			
Branco	10 (55,56)	8 (44,44)	0,061^*^
Preto/Pardo	32 (80,00)	8 (20,00)	
Empregabilidade			
Sim	34 (70,83)	14 (29,17)	0,446^*^
Não	8 (80,00)	2 (20,00)	
Atua como profissional do sexo??			
Sim	32 (78,05)	9 (21,95)	0,120^*^
Não	10 (58,82)	7 (41,18)	
Renda mensal (salário-mínimo)			
Até 1	17 (73,91)	6 (26,09)	0,546^*^
> 1	25 (71,43)	10 (28,57)	

* Teste Qui-quadrado;

† Teste Exato de Fisher.

Evidenciou-se maior proporção de níveis depressivos moderado/grave em indivíduos que relataram ter sofrido violência na espacialidade dos serviços de saúde (p=0,030) (Tabela 4).

**Tabela 4:** Violências contra travestis e transexuais segundo níveis depressivos (n=58), Natal, Rio Grande do Norte, Brasil, 2023

Violências	Níveis depressivos		Valor de *p*
	Mínima/ leve n (%)	Moderado/Grave n (%)	
Já sofreu violência			
Sim	41 (73,21)	15 (26,79)	0,488^*^
Não	1 (50,00)	1 (50,00)	
Violência física			
Sim	17 (73,91)	6 (26,09)	0,845^*^
Não	25 (71,43)	10 (28,57)	
Violência verbal			
Sim	35 (71,43)	14 (28,57)	0,655^*^
Não	7 (77,78)	2 (22,22)	
Violência sexual			
Sim	22 (64,71)	12 (35,29)	0,121^‡^
Não	20 (83,33)	4 (16,67)	
Violência psicológica			
Sim	18 (66,67)	9 (33,33)	0,363^*^
Não	24 (77,42)	7 (22,58)	
Quantidade de tipos de violências sofrida			
Até 1	15 (93,75)	1 (6,25)	0,253^‡^
≥2	27 (64,29)	15 (35,71)	
Espacialidade da violência - casa			
Sim	15 (71,43)	6 (28,57)	0,891^*^
Não	27 (72,97)	10 (27,03)	
Espacialidade da violência - Rua			
Sim	29 (67,44)	14 (32,56)	0,159^*^
Não	13 (86,67)	2 (13,33)	
Espacialidade da violência - Escola			
Sim	18 (72,00)	7 (28,00)	0,952^*^
Não	24 (72,73)	9 (27,27)	
Espacialidade da violência - Serviços de saúde			
Sim	3 (42,86)	4 (57,14)	0,030^†^
Não	39 (76,47)	12 (23,53)	
Espacialidade da violência - Outros serviços públicos e privados			
Sim	4 (66,67)	2 (33,33)	0,660^*^
Não	38 (73,08)	14 (26,92)	
Agressor era familiar?			
Sim	15 (71,43)	6 (28,57)	0,891^†^
Não	27 (72,97)	10 (27,03)	
Por qual tipo de agressor sofreu violência?			
Conhecido	16 (72,73)	6 (27,27)	0,251^‡^
Desconhecido	17 (80,95)	4 (19,05)	
Conhecido e desconhecido	7 (53,85)	6 (46,15)	
Não sofreu agressão	2 (100,00)	0 (0,00)	

* *Teste Qui-quadrado; †Teste Exato de Fisher; ‡Razão de verossimilhança.*

Na análise múltipla, as variáveis que permaneceram associadas aos níveis depressivos foram: estado civil e experiência de violência no espaço dos serviços de saúde. Os participantes que eram solteiros apresentaram maior prevalência de níveis depressivos (moderado/grave) quando comparados aos indivíduos vivendo com companheiro (RP=1,19). Do mesmo modo, também se evidenciou maior prevalência de níveis depressivos moderado/grave (RP=2,30) em quem afirmou ter sofrido violência no espaço dos serviços de saúde (Tabela 5).

**Tabela 5:** Modelo de regressão múltipla sobre a associação entre níveis depressivos, histórico de violência e estado civil entre travestis e transexuais assistidas por organizações não governamentais no estado do Rio Grande do Norte, Natal, Rio Grande do Norte, Brasil, 2023

Variáveis	RP^*^ bruta (IC^†^ 95%)	RPa (IC 95%)
Estado civil		
Vivendo com companheiro	1^‡^	
Solteiro	1,27 (1,19-1,76)	1,19 (1,10-1,28)
Espacialidade da violência - Serviço de Saúde		
Não	1^‡^	
Sim	1,30 (1,03-1,79)	2,30 (1,10-4,81)

* *Razão de Prevalência/Razão de Prevalência ajustada; † Intervalo de Confiança; ‡ Categoria de referência.*

## DISCUSSÃO

Esta pesquisa evidenciou alta prevalência de níveis depressivos moderados a graves em pessoas transexuais e travestis assistidas por Organizações Não Governamentais (ONGs) do Rio Grande do Norte, Brasil. Esta prevalência esteve associada ao estado civil e à experiência de violência no contexto dos serviços de saúde.

A elevada magnitude na prevalência (27,6%) de sintomas depressivos moderados e graves nos 15 dias que antecederam a coleta de dados foi maior do que em estudos desenvolvidos no Camboja^(5)^, onde 21% dos participantes apresentaram sintomas graves de depressão; e no Brasil^(13)^, onde 16,0% vivenciaram depressão severa. Esses achados podem ser influenciados por diferenças nos instrumentos de mensuração das variáveis. Contudo, é notável a intensidade dos níveis depressivos na vida de travestis e transexuais em diferentes territórios.

A prevalência de sintomas depressivos na população transgênero é significativamente maior em relação aos seus pares cisgênero, com uma razão de prevalência 1,03 vezes maior para o desenvolvimento de depressão e 2,53 para tentativa de suicídio, em comparação com indivíduos cisgênero^(18)^.

Essas diferenças podem ser explicadas pela *Gender Minority Stress Theory*, que sugere que minorias sociais vivenciam estressores específicos na sociedade, adicionais aos estressores cotidianos. Nesse contexto, pessoas trans enfrentam alta prevalência de estressores, como assédio, trauma, rejeição e abandono relacionados ao gênero, além das inúmeras violências às quais estão expostas na sociedade, manifestadas através da transfobia^(8,19)^.

O fenômeno da depressão entre travestis e transexuais parece ser resultado de um contexto de vida onde o debate sobre questões de gênero e sexualidade é limitado, apesar de emergente. Os efeitos nocivos são perceptíveis na dificuldade de ajustamento social, nos relacionamentos intrafamiliares e na escassez de oportunidades no mercado de trabalho^(5)^. Neste cenário, o presente estudo mostrou que a maioria das travestis e transexuais recebia até um salário mínimo e não tinha renda fixa. Associado ao menor acesso ao trabalho e renda, as participantes deste estudo apresentaram alta prevalência de experiência violenta (96,60%), especialmente de violência verbal e sexual, o que pode estar relacionado com a alta prevalência de sintomas depressivos moderados a graves observada nesta população.

A violência dirigida aos corpos trans faz parte da estrutura social brasileira, associada ao machismo e à heterocisnormatividade, que agem de maneira insidiosa e operam por meio de lógicas que hierarquizam corpos e vidas. Nessa lógica, as pessoas trans são vulnerabilizadas e oprimidas por não corresponderem aos padrões hegemônicos instituídos. Sob essa mesma égide, podem ocorrer assassinatos, estupros, agressões físicas, verbais, psicológicas, sexuais, ataques em ambientes públicos ou privados, em serviços de saúde e instituições de ensino^(20)^.

No tópico das relações sociais prejudicadas, encontramos neste estudo que pessoas travestis e transexuais solteiras são mais propensas a desenvolver sintomas depressivos moderados a graves quando comparadas àquelas que têm companheiro(a). Essa realidade denota a dificuldade de pessoas travestis e transexuais em manterem relacionamentos estáveis, pois são, socialmente, marginalizadas nos encontros afetivos. Deve-se levar em consideração que a afetividade humana tem relação direta com a saúde mental, e a marginalização neste campo pode levar os sujeitos a problemas na autoestima e no autoconceito, manifestando-se em sintomas depressivos^(21)^.

Em contrapartida, medidas que favoreçam o reconhecimento de suas identidades de gênero, a educação de gênero no sistema educacional e a aceitação familiar e social, constituem-se em estratégias para melhorar a sociabilidade das pessoas travestis e transexuais e preservar sua saúde mental. O suporte social, também caracterizado pelos relacionamentos conjugais/afetivos positivos, é reconhecido como um fator protetivo contra a ideação suicida, por exemplo, somado à vivência entre pares, disponibilidade de serviços de atenção à saúde, consistência disciplinar e coesão familiar^(22-23)^.

A experiência de violência nos serviços de saúde associou-se a uma maior prevalência de sintomas depressivos moderados a graves. O despreparo dos profissionais de saúde para lidar com a diversidade sexual e de gênero é um tema amplamente discutido na literatura. Geralmente, pessoas trans consideram que suas necessidades não foram devidamente atendidas; a assistência foi percebida como inadequada e/ou sofreram preconceito por parte de algum membro da equipe de saúde^(24-26)^. Essa situação é repetida e documentada em diversos estudos, os quais relacionam a violência por parte dos profissionais de saúde como um forte fator de risco para depressão^(5,26-27)^.

Outra problemática que surge com as altas prevalências de sintomas depressivos na população trans é o comportamento suicida, que, apesar de não ter sido abordado neste estudo, é importante ressaltar que a ideação e a tentativa de suicídio também apresentam alta prevalência entre travestis e transexuais. A ideação suicida está associada a sintomas moderados e graves de depressão nessa população^(28)^.

Assim, entende-se que, além de sintomas depressivos e seus fatores associados, travestis e transexuais estão sujeitas a mortes voluntárias e involuntárias, quando condicionadas às violências impostas nos múltiplos contextos sociais, institucionais e familiares. No contexto brasileiro, a realidade apresentada contrapõe-se à Política Nacional de Saúde Integral de Lésbicas, Gays, Bissexuais, Travestis e Transexuais (PNSI-LGBT)^(15)^, que fornece diretrizes para a sensibilização dos profissionais de saúde sobre os direitos dessas pessoas, por meio de estratégias de educação permanente em saúde e serviços especializados para um atendimento adequado e o enfrentamento aos problemas de saúde mental.

### Limitações do estudo

Este estudo apresenta algumas limitações que devem ser consideradas ao interpretar os resultados. Primeiramente, a natureza transversal da pesquisa impede a inferência de relações causais entre os fatores estudados e os níveis depressivos observados. Além disso, a amostra, recrutada em apenas quatro Organizações Não Governamentais, limita a generalização dos resultados para outras regiões e contextos. Outro aspecto importante é a dependência do autorrelato para a coleta de dados, que pode estar sujeita a vieses de memória e desejabilidade social. Ademais, o cenário do estudo, as ONGs, e a identidade organizativa de classe podem ter conferido um efeito protetivo contra o desenvolvimento de sintomas depressivos, sendo possível que, entre pessoas travestis e transexuais não assistidas por ONGs, a prevalência seja mais alarmante. Por fim, a utilização de instrumentos específicos para a medição de sintomas depressivos pode não capturar completamente a complexidade e a nuance das experiências de saúde mental dessa população, especialmente em um contexto culturalmente diverso como o brasileiro.

### Contribuições para a área da Enfermagem, saúde ou política pública

Este estudo contribui para a área da saúde ao observar uma população-chave e vulnerável ao processo de adoecimento psíquico com determinação social. A situação de saúde dessa população pode orientar profissionais de saúde na identificação dos fatores relacionados ao estresse de minorias, no uso de ferramentas e dispositivos sociais para proteção e preservação da saúde, e na defesa pública e junto aos legisladores pelos direitos das pessoas travestis e transexuais. Por fim, a Política Nacional de Saúde Integral de Lésbicas, Gays, Bissexuais, Travestis e Transexuais (PNSI-LGBT) é desafiada a ampliar o modo de produzir cuidados às pessoas travestis e transexuais para reduzir o adoecimento psíquico, enfrentar a incidência das violências nos serviços de saúde e promover saúde mental. Dessa forma, as contribuições desse estudo têm o potencial de influenciar positivamente várias facetas da Enfermagem, da saúde pública e das políticas públicas, promovendo uma sociedade mais inclusiva e justa.

## CONCLUSÕES

Evidenciamos níveis elevados de depressão (moderados a graves) na população travesti e transexual assistida por organizações não governamentais, associados ao estado civil e à violência nos serviços de saúde. Nosso estudo ressalta a importância de políticas públicas mais robustas e programas de formação para profissionais de saúde que enfatizem a diversidade, inclusão e competência cultural. Além disso, reitera a necessidade de iniciativas de advocacia e apoio social que abordem a marginalização, a discriminação e a violência enfrentadas por esses indivíduos. Recomenda-se a realização de estudos brasileiros com outros recortes metodológicos, como pesquisas qualitativas e teóricas ou de base populacional, de modo a produzir outros níveis de evidência.
